# An HMM approach expands the landscape of sesquiterpene cyclases across the kingdom Fungi

**DOI:** 10.1099/mgen.0.000990

**Published:** 2023-04-19

**Authors:** Hayat Hage, Julie Couillaud, Asaf Salamov, Margot Loussouarn-Yvon, Fabien Durbesson, Elena Ormeño, Sacha Grisel, Katia Duquesne, Renaud Vincentelli, Igor Grigoriev, Gilles Iacazio, Marie-Noëlle Rosso

**Affiliations:** ^1^​ INRAE, Aix Marseille Univ, UMR1163, Biodiversité et Biotechnologie Fongiques, Marseille, France; ^2^​ Aix Marseille Univ, CNRS, Centrale Marseille, iSm2, Marseille, France; ^3^​ US Department of Energy Joint Genome Institute, Lawrence Berkeley National Laboratory, Berkeley, CA 94720, USA; ^4^​ AFMB, UMR CNRS 7257, USC 1408, Marseille, France; ^5^​ CNRS, Aix Marseille Univ, IRD, Avignon Univ, IMBE, Marseille, France; ^6^​ Environmental Genomics and Systems Biology, Lawrence Berkeley National Laboratory, Berkeley, CA 94720, USA; ^7^​ Department of Plant and Microbial Biology, University of California Berkeley, Berkeley, CA, USA; ^†^​ Present address: Bioaster, Lyon, France; ^‡^​ Present address: Department of Biology and Biological Engineering, Systems and Synthetic Biology Division, Chalmers university of Technology, Gothenburg, Sweden

**Keywords:** Keywords : fungi, genome, Polyporales, sesquiterpene, terpene synthase

## Abstract

Sesquiterpene cyclases (STC) catalyse the cyclization of the C15 molecule farnesyl diphosphate into a vast variety of mono- or polycyclic hydrocarbons and, for a few enzymes, oxygenated structures, with diverse stereogenic centres. The huge diversity in sesquiterpene skeleton structures in nature is primarily the result of the type of cyclization driven by the STC. Despite the phenomenal impact of fungal sesquiterpenes on the ecology of fungi and their potentials for applications, the fungal sesquiterpenome is largely untapped. The identification of fungal STC is generally based on protein sequence similarity with characterized enzymes. This approach has improved our knowledge on STC in a few fungal species, but it has limited success for the discovery of distant sequences. Besides, the tools based on secondary metabolite biosynthesis gene clusters have shown poor performance for terpene cyclases. Here, we used four sets of sequences of fungal STC that catalyse four types of cyclization, and specific amino acid motives to identify phylogenetically related sequences in the genomes of basidiomycetes fungi from the order Polyporales. We validated that four STC genes newly identified from the genome sequence of *Leiotrametes menziesii*, each classified in a different phylogenetic clade, catalysed a predicted cyclization of farnesyl diphosphate. We built HMM models and searched STC genes in 656 fungal genomes genomes. We identified 5605 STC genes, which were classified in one of the four clades and had a predicted cyclization mechanism. We noticed that the HMM models were more accurate for the prediction of the type of cyclization catalysed by basidiomycete STC than for ascomycete STC.

## Data Summary

All STC sequences used in this study were retrieved from the DOE JGI’s Mycocosm repository (https://mycocosm.jgi.doe.gov/mycocosm/home).

The HMM models are available at https://doi.org/10.57745/LORDNA.

**Impact Statement**

Fungi produce a vast variety of sesquiterpenes and derived terpenoids, including notorious bioactive mycotoxins, cytotoxins and precursors of anticancer molecules. The diversity of the structure of fungal sesquiterpene skeletons relies on STC that catalyse the conversion of linear farnesyl diphosphate into mono- or polycyclic terpenes. Recent phylogenetic analyses on characterized fungal STC suggested that STC that catalyse a same primary cyclization type originated from a common ancestor gene. Here, we enlarged the set of STC with predicted cyclization type by searching for homologues in the genome of 24 Polyporales fungi. We used these four enlarged sets of sequences to build four HMM models suitable for identifying STC from the whole fungal kingdom. The biochemical characterization of four newly identified STC confirmed that the four HMM models can be used to predict the structure of the terpene skeletons produced by fungal basidiomycete STC.

## Introduction

Terpene cyclases (sometimes named terpene synthases) catalyse the cyclization of polyprenyl linear precursors into a range of mono- or polycyclic structures, providing the primary step towards the extraordinary diversity of terpenes [1]. The polyprenyl precursors are geranyl diphosphate (GPP; the C10 precursor of monoterpenoids), farnesyl diphosphate (FPP; the C15 precursor of sesquiterpenoids) or geranylgeranyl diphosphate (GGPP; the C20 precursor of diterpenoids). In the case of sesquiterpene cyclases (STC), the removal of inorganic diphosphate (PPi) from (2*E*,6*E*)-FPP generates a reactive carbocation. Further 1,10 or 1,11 cyclization generates a *E*,*E*-germacradienyl cation or trans-humulyl cation, respectively. Alternatively, an initial isomerization of (2*E*,6*E*)-FPP may form (3*R*)-nerolidyl pyrophosphate (NPP), further modified via 1,6; 1,7; 1,10 or 1,11 cyclization, leading to a bisabolyl, cycloheptanyl, (*Z*,*E*)-germacradienyl or (*Z*,*E*)-humulyl cation, respectively [2]. The resulting carbocations can undergo further cyclization reactions, hydride transfers or methyl migrations, increasing the diversity of carbon skeletons that can be generated by STC [3].

In fungi, the STC, which catalyse the cyclization of FPP, are more abundant than mono- and di-terpene cyclases, consistent with fungi being prolific producers of sesquiterpenes and sesquiterpenoids [4]. About a hundred fungal STC have been characterized to date. In ascomycetes, efforts were mainly focused on enzymes involved in the synthesis of mycotoxins, such as trichodiene synthases [5–9], aristolochene synthases [10, 11], or the synthesis of phytotoxins, such as β-caryophyllene, α-humulene or botrydial [12, 13]. In addition, a few STC have been characterized from endophyte ascomycetes, for example to assess their relevance in the production of mycodiesel [14]. In basidiomycetes, recent efforts have been largely dedicated to the STC involved in the synthesis of **Δ**6-protoilludene, a precursor of anti-cancer molecules, for example in edible fungi [15], in tree pathogens [15, 16] or in wood decay fungi [17–19], among others. Other studies have scrutinized the role of STC in the settlement of endophytic fungi in the plant host [20]. These analyses indicated that, in the vast majority of cases, fungal STC catalyse the production of a major sesquiterpene product and have limited promiscuity [21].

Yet, much is still to be done for accurate identification of fungal STC and their biochemical characterization. Genome mining for terpene cyclases in fungi is limited by the low sequence similarity between plant and microbial enzymes outside the metal binding motifs [D(D/E/N)XX(D/E)] and NSE/DTE [15]. Contrary to plant STC sequences, which contain a highly conserved 252 aa-long N-terminal region, the fungal STC lack this conserved N-terminal region and the C-terminal region shows low sequence identity with the plant sequences. Consequently, the Hidden Markov Models (HMM models) classified in the Pfam database, which were built from plant terpene cyclases, are not optimal for the identification of fungal STC (e.g. PF01397). The domains related to magnesium ion binding (e.g. PF03936, IPR005630) and anti-parallel α-helices (e.g. IPR008949 and IPR034686) are not discriminant between the polyprenyl transferases that synthesize the linear isoprenoid chains and the terpene cyclases. Recently, the Pfam domain PF19086, for terpene synthase family 2, C-terminal metal binding, was added to the Pfam database [22]. Other Pfam domains include PF06330, IPR024652 and IPR010458, identified from trichodiene synthases. However, the tools based on the identification of biosynthesis gene clusters have shown limited success for fungal genome mining [23, 24].

Interestingly, previous analyses on fungal STC suggested that phylogenetically related enzymes produce terpenes with a same skeleton structure [15, 19, 25, 26]. From these studies, several STC clades were identified, among which clade one contained STC predicted to catalyse the 1,10-cyclization of (2*E*,6*E*)-FPP carbocation, clade two contained STC predicted to catalyse the 1,10-cyclization of the (3*R*)-NPP carbocation, clade three contained STC predicted to catalyse the 1,11 cyclization of (2*E*,6*E*)-FPP carbocation (trans-humulyl-type cyclases), and clade four contained STC predicted to catalyse the 1,6 or 1,7 cyclization of the (3*R*)-NPP carbocation [17].

In this work, we have developed a new method for the identification of STC genes in fungal genomes. Using a set of characterized STC with known cyclization type, we searched for homologs in the basidiomycetes Polyporales and expanded the set of sequences with 309 additional candidate STC. We validated that four STC from the fungus *Leiotrametes menziesii*, each classified in a different clade, catalysed the expected cyclization of FPP.

We used this enlarged set of predicted STC to build a first generation-HMM model for each cyclization type. After mining 656 fungal genomes, we used the STC sequences that had the highest scores with the first generation-HMM models to build more robust second generation-HMM models. The second generation-HMM models allowed the identification of 5605 candidate STC genes across the kingdom Fungi, among which 4194 candidate STC genes across Basidiomycota, which were attributed to a prediction for the mechanism of cyclization. Our results show that a significant proportion of STC families in Basidiomycota possibly result from tandem duplication events.

## Methods

### Classification of Polyporales STC genes according to the predicted structure of the terpene skeleton

**Fig. 1.**
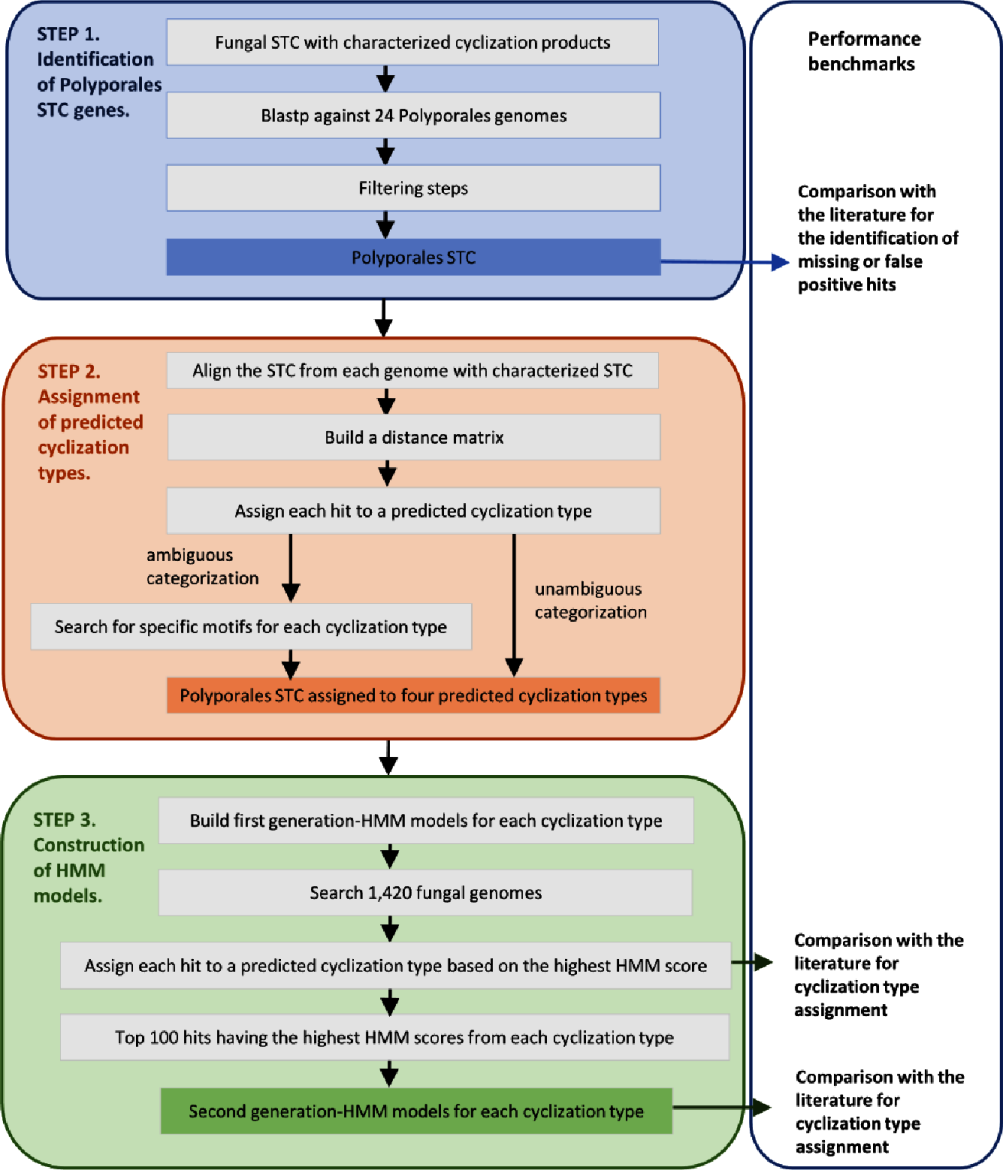
Rationale for the construction of HMM models for fungal sesquiterpene cyclases (STC).

Candidate STC coding genes were retrieved from 24 Polyporales genomes using blastp search (version 2.7) and filters as described in Fig. 1 and in the Results section. To classify the newly identified Polyporales STC genes according to each of the four cyclic structures, we first aligned the Polyporales STC protein sequences with the sequences of characterized STC using MAFFT [27]. We computed distance matrices using Fprotdist from the phylip package in EMBOSS, which considers the categorization of the amino acids according to their physicochemical properties. The dendrograms were built from those matrices using the ‘dendextend’ package in R [28]. We obtained four sets of sequences, with the predicted cyclization types 1, 2, 3 and 4, in addition to a fifth set called ‘unclassified’, which contained the Polyporales STC sequences that did not cluster unambiguously with any of the characterized STC. We used the four sets of classified STC sequences to search for signature motifs of each cyclization type using the MERCI tool [29]. This tool identifies highly represented motifs in a set of sequences (here the sequences of STC with a specific cyclization type, considered as ‘positive’ sequences), that are absent in the other sets (here the sequences of STC from the three other cyclization types, considered as ‘negative’ sequences). The phylogenetic analyses of STC sequences were done using Randomized Axelerated Maximum Likelihood (RAxML) with PROTGAMMAAUTO model and 500 bootstrap values.

### Generation of HMM-models for the identification and classification of fungal STC

The STC sequences that produce a same predicted cyclic carbocation were aligned using MAFFT version v7.429, with the option GOP 2.3 and GEP 0.63 [27]. The poorly aligned regions were removed using trimAl 1.2 [30] and the quality of each alignment was manually inspected. Each of the four trimmed alignments was used to build HMM Hidden Markov Model using the ‘hmmbuild’ command from HMMER3.1 [31]. The HMM models attributed to each cyclization type were called cladeX-HMM, where ‘X’ is the name of the clade corresponding to the cyclization type. Those four cladeX-HMM models (here named first generation-HMM models) served to search for STC hits in all fungal genomes available in Mycocosm (656 genomes on 1 April 2019), using the hmmsearch command with gathering threshold=25. A hit was attributed to a cyclization type according to the cladeX-HMM model that found the hit. If a hit was commonly found by more than one cladeX-HMM, we assigned it to the cyclization type of the model with the highest HMM score. Using this approach, Polyporales STC sequences were largely overrepresented in the set of sequences used to build first generation-HMM models. In order to remedy potential bias in these HMM models, we gathered for each cyclization type the candidate STC retrieved from basidiomycete and ascomycete genomes. From each set, the 100 sequences with the highest HMM scores were aligned together as described previously and the trimmed alignments were used to build second generation-cladeX-HMM models.

### Activity assays on four newly identified fungal STC

We selected four newly identified STC from *Leiotrametes menziesii* CIRM-BRFM 1781 for activity assays, with Mycocosm accession numbers Prot ID #671 455 (LmSTC1), #931 910 (LmSTC2), #920 014 (LmSTC3) and #1 051 559 (LmSTC4). The synthetic coding sequences of (LmSTC1) and (LmSTC3) were obtained from NZytech and cloned under the T7 promoter in the pHTP1 vector (NZYTech) with a N-terminal 6His-tag fusion. The synthetic coding sequences for LmSTC4 and LmSTC2 were obtained from Twist Bioscience and cloned under the T7 promoter in a modified pET28 vector with a N-terminal 6His-tag fusion. Transformed Rosetta (DE3) *Escherichia coli* cells were grown overnight in Luria-Bertani (LB) medium at 37 °C with 220 r.p.m. agitation, and 1/50^e^ pre-culture was transferred into terrific broth (TB) medium for 2 h at 37 °C, then at 16 °C in the presence of IPTG until OD_600_ reached 6–10. After centrifugation at 4 000 ***g***, the cells were suspended in lysis buffer (Tris-HCl 50 mM, NaCl 150 mM, pH 7.5, lysozyme 1 g l^–1^ final) and the suspension was frozen at −20 °C. After thawing, 2 µl of benzonase were added and the samples were incubated at 4 °C for 30 min.

The cells were broken with a French Press at 1.3 kbar and the samples were centrifuged for 20 min at 6 000 ***g***, 4 °C. The supernatants were loaded on agarose Ni-NTA resin and incubated at 4 °C, for 1 h with gentle agitation. The proteins were eluted with a 50 mM - 250 mM imidazole gradient in 50 mM NaH_2_PO_4_, 300 mM NaCl. After buffer exchange on Sephadex G-25, the proteins were eluted in 50 mM Tris-HCl, 5 mM MgCl_2_, pH 7.5 before analysis on 4–12 % SDS-PAGE electrophoresis gel. Alternatively, the enzymes were purified on a 5 ml Ni His-trap column (Cytiva) with an ÄKTA Purifier (Cytiva) with 250 mM imidazole in Tris-HCl 50 mM, NaCl 150 mM, pH7.5. The buffer exchange was done by gel filtration on a Hiload 16/600 Superdex 75 pg column (Sigma-Aldrich) in 50 mM Tris-HCl, 5 mM MgCl_2_, pH7.5. The enzymes were stored at 4 °C until activity assays.

The bioconversion assays were performed on (*E*,*E*)-farnesyl diphosphate (FPP), geranyl diphosphate (GPP) and geranylgeranyl diphosphate (GGPP) 5 mM final, in 50 mM Tris-HCl, 5 mM MgCl_2_, pH 7.5, in 500 µl reaction volume, at 37 °C for 24 h with gentle agitation. For controls, either the enzyme or substrate were omitted. The reaction products were extracted with 300 µl of diethyl ether. After 1 min vortex and 60 s centrifugation at 12500 r.p.m., the organic phase was collected and the remaining water was removed with addition of a pinch of anhydrous sodium sulphate and incubation at room temperature during 15 min. The samples were centrifuged for 60 s at 12500 r.p.m. and the organic phase was transferred to a gas chromatography (GC) vial. The reaction products were separated on a ZB-WAXplus (30 m L × 0.53 mm ID × 1.00 µm df) column (Zebron/Phenomenex) with a Shimadzu GC-2014 instrument. The system was operated with a constant pressure mode (50 kPa) with nitrogen carrier gas and the column flow was set at 1134 ml min^−1^. The GC inlet temperature was set to 250 °C. The injection volume was 1 µl and the split ratio was 7.9. The GC oven profile was set to start at 55 °C (held for 4 min) and then increased to 190 °C at a rate of 7 °C min^−1^ (held for 30 min). The column flow was set at 3.61 ml min^−1^ and the temperature for the FID detector was 250 °C.

### Identification of fungal STC products

The sesquiterpene analyses were performed using GC (Gas Chromatography, Hewlett Packard GC6890) coupled to a Mass Selective Detector (MSD, HP 5973 N) in splitless mode. Terpene separation was achieved in a HP-5MS capillary column (30 m × 250 μm × 0.25 µm, length, external diameter and internal diameter), in constant flow mode (1.2 ml min^−1^). One microlitre of each extracted sample was injected through an automatic injector (ALS 7683). Helium (99.995 %) was used as carrier gas. The oven temperature was initially set at 80 °C for 2 min and then increased to 200 °C at a rate of 5 °C, then increased to 260 °C at a rate of 20 °C min^−1^. It then remained constant for 5 min. The parameters of the MSD for electron impact (EI) mode were: ion source: 230 °C; MS quadrupole: 150 °C; electron energy: 70 eV; electron multiplayer energy: 1200 V. Data were acquired in scan mode from 40 to 500 amu. The identity of terpenes was established by comparison of their retention time and mass spectrum to those of generated libraries of retention indexes (NIST14). To calculate terpene concentrations, the integrated area of each peak was multiplied by the average response factor of terpene standards (Aldrich–Firmenich, Sigma), and divided by the sample volume.

For Lm-STC3, the enzymatic reaction was conducted in a 250 ml round bottom flask in Tris-HCl 300 mM, 5 mM MgCl2, pH 7.5, with 5 mM FPP and 0.2 U ml^−1^ of enzyme (final volume 100 ml) at 40 °C under magnetic agitation. After 24 h, the reaction was extracted with 3×100 ml of pentane, the organic phases combined, dried over anhydrous Na_2_SO_4_, filtrated and the solvent evaporated under reduced pressure. The residue was then purified by flash chromatography over silica with pentane as eluant. Fractions containing Δ−6 protoilludene were combined, evaporated and analysed by NMR. The NMR spectra were in full accordance with those reported for chemically synthesized Δ−6 protoilludene [32].

## Results and discussion

### Overall strategy for the construction of HMM models for fungal STC

The overall strategy to build HMM models for the four clades of fungal STC is presented in Fig. 1.

### Identification of Polyporales STC and classification according to the predicted structure of the carbocation they produce

We first selected nine fungal STC, for which the structure of the reaction product on FPP had been characterized (Table S1, available in the online version of this article). For the 1,10-cyclization of (2*E*,6*E*)-FPP (clade 1), the selected sequences were Cop2 from *Coprinopsis cinerea* and Omp3 from *Omphalotus olearius*. For the 1,10-cyclization of the (3*R*)-NPP (clade 2), the selected sequences were the gene model ProtID #128 017 (Mycocosm, Joint Genome Institute) from *Stereum hirsutum* FP-91666 SS1 v1.0 and Omp5a. For the 1,11 cyclization of (2*E*,6*E*)-FPP (clade 3), the selected sequences were BcBOT2 from *Botrytis cinerea* and Omp6. Finally, for the 1,6 or 1,7 cyclization of the (3*R*)-NPP (clade 4), the selected sequences were Ffsc6 from *Fusarium fujikuroi*, Fompi84944 from *Fomitopsis pinicola* and Omp10. Using a blastp search against 24 newly sequenced fungal genomes ([33]; Table S2), we identified a total of 454 candidate STC protein sequences that had more than 30 % identity and 60 % coverage with the sequences of the characterized enzymes (Table 1). On average, we retrieved nine candidate STC per genome using Basidiomycota sequences as query, and two candidate STC per genome using Ascomycota sequences as query (Table S3). From those, 318 genes had a domain related to terpene synthases as determined by SUPERFAMILY HMM search (https://supfam.mrc-lmb.cam.ac.uk/SUPERFAMILY/) and classified in SCOP 48576, which includes the following five families: isoprenyl diphosphate synthase (SCOP 48577), squalene synthase (SCOP 48580), terpenoid cyclase C-terminal domain (SCOP 48583), aristolochene/pentalenene synthase (SCOP 48586) and trichodiene synthase (SCOP 69113). Finally, we filtered the genes for the presence of the conserved metal-binding motives [(D/N)DXX(D/E) or DDXXXE] in the N-terminal region and (NxxxSxxxE) in the C-terminal region, and a sequence length comprised between 250 and 500 amino acids. In total, we retrieved 309 genes as candidate STC genes. To ensure that those filtering steps did not produce false positives and that no hits were missing, we tested the method on the genome of *Omphalotus olearius* Ompol1. As expected, we retrieved from this genome the eleven STC genes previously identified by Wawrzyn *et al*. [17] from which two were removed during the filtering steps.

**Table 1.** Counts of candidate sesquiterpene cyclase genes identified in each of the 24 Polyporales genomes, obtained after three filtering steps

Genome	no. of blastp hits	Filter 1 Sequence identity >30 % and query coverage >60 %	Filter 2 SUPERFAMILY SCOP 48576 and metal binding domains	Filter 3 250<length (aa) <500
Abobie1	133	17	11	10
Artel1	125	19	11	11
Earsca1	178	23	21	20
Fomfom1	147	20	16	16
Fomros1	122	23	17	17
Hexnit1	153	22	16	16
Irplac1	96	13	6	4
Leisp1	176	28	20	20
Pipbet1	118	26	19	17
Polbr1	117	14	11	11
Pycci1	116	10	8	8
Pycco1	110	16	9	9
Pycpun1	119	18	8	8
Pycsa1	135	20	13	13
Trabet1	124	20	13	13
Trace1	80	15	11	11
Traci1	118	17	11	10
Tragib1	126	19	12	12
Tralac1	157	18	15	14
Tralj1	123	17	11	10
Tramax1	122	17	12	12
Tramen1	167	24	18	18
Tramey1	131	18	14	14
Trapol1	133	20	15	15
**Total**	**3126**	**454**	**318**	**309**

**Fig. 2.**
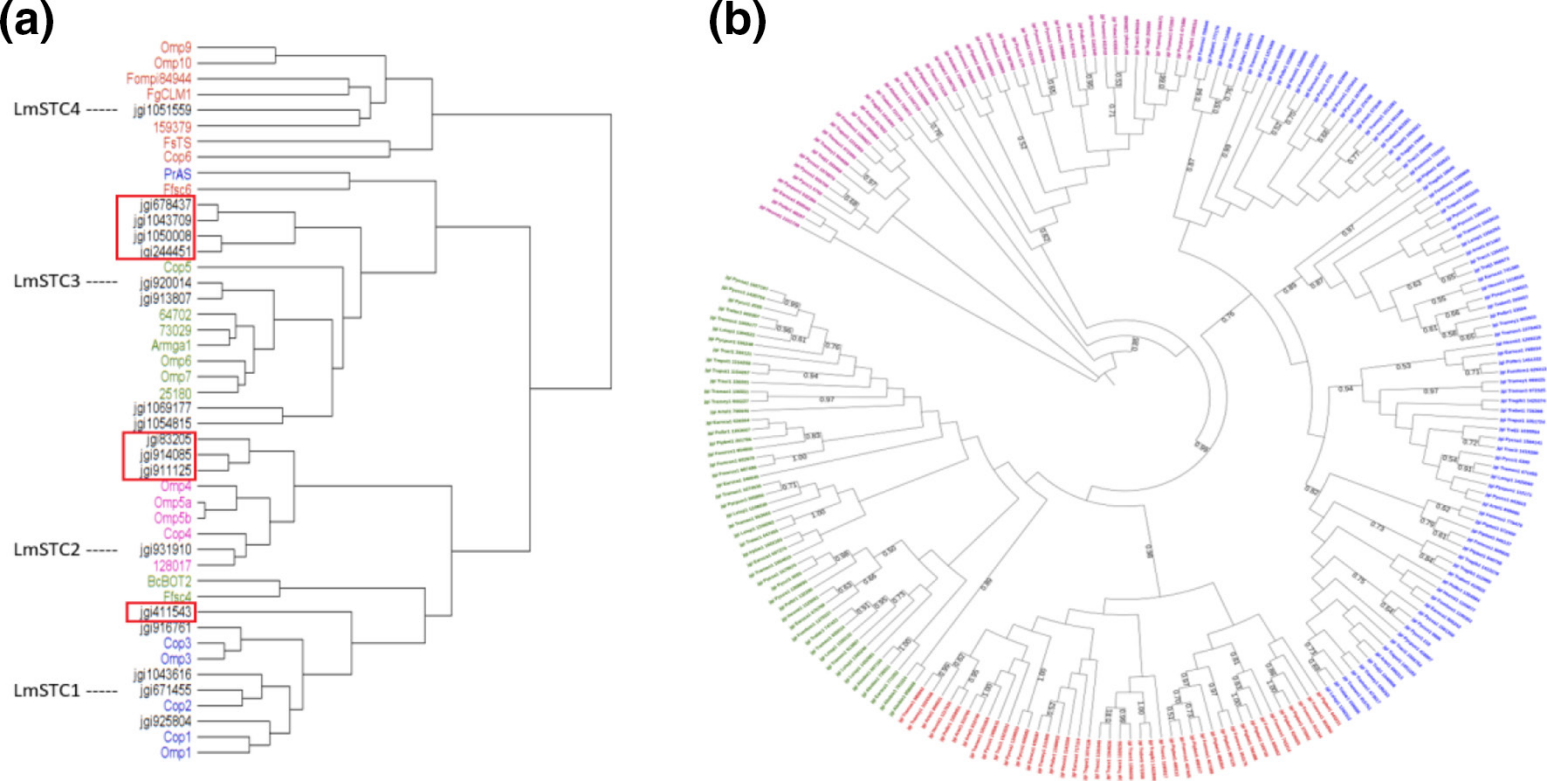
Classification of newly identified sesquiterpene cyclases from 24 Polyporales species. a) Example of a dendrogram built from a Fprotdist distance matrix including the STC sequences identified in *Leiotrametes menziesii* (in black) and the characterized STC from clade 1 (blue), clade 2 (pink), clade 3 (green) and clade 4 (red). The identifiers for protein sequences are those from the Mycocosm database at JGI and are listed in Table 1. The sequences with ambiguous clade assignment are highlighted by a red box. **b**) RAxML phylogenetic tree of 221 Polyporales STC sequences classified without ambiguity into a clade.

We next classified the 309 candidate STC according to the predicted structure of the carbocation they produce. First, the candidate STC from each Polyporales genome were compared to the sequence of characterized enzymes using Fprotdist distance matrices (Fig. 2a). Out of the 309 Polyporales STC, we classified 87 candidate STC in clade 1, 45 in clade 2, 48 in clade 3 and 41 in clade 4. Our phylogenetic analysis confirmed the phylogenetic relationship of the Polyporales sequences classified in clades 1, 3 and 4 (Fig. 2b). Yet, 88 candidate STC could not be unambiguously classified.

**Fig. 3.**
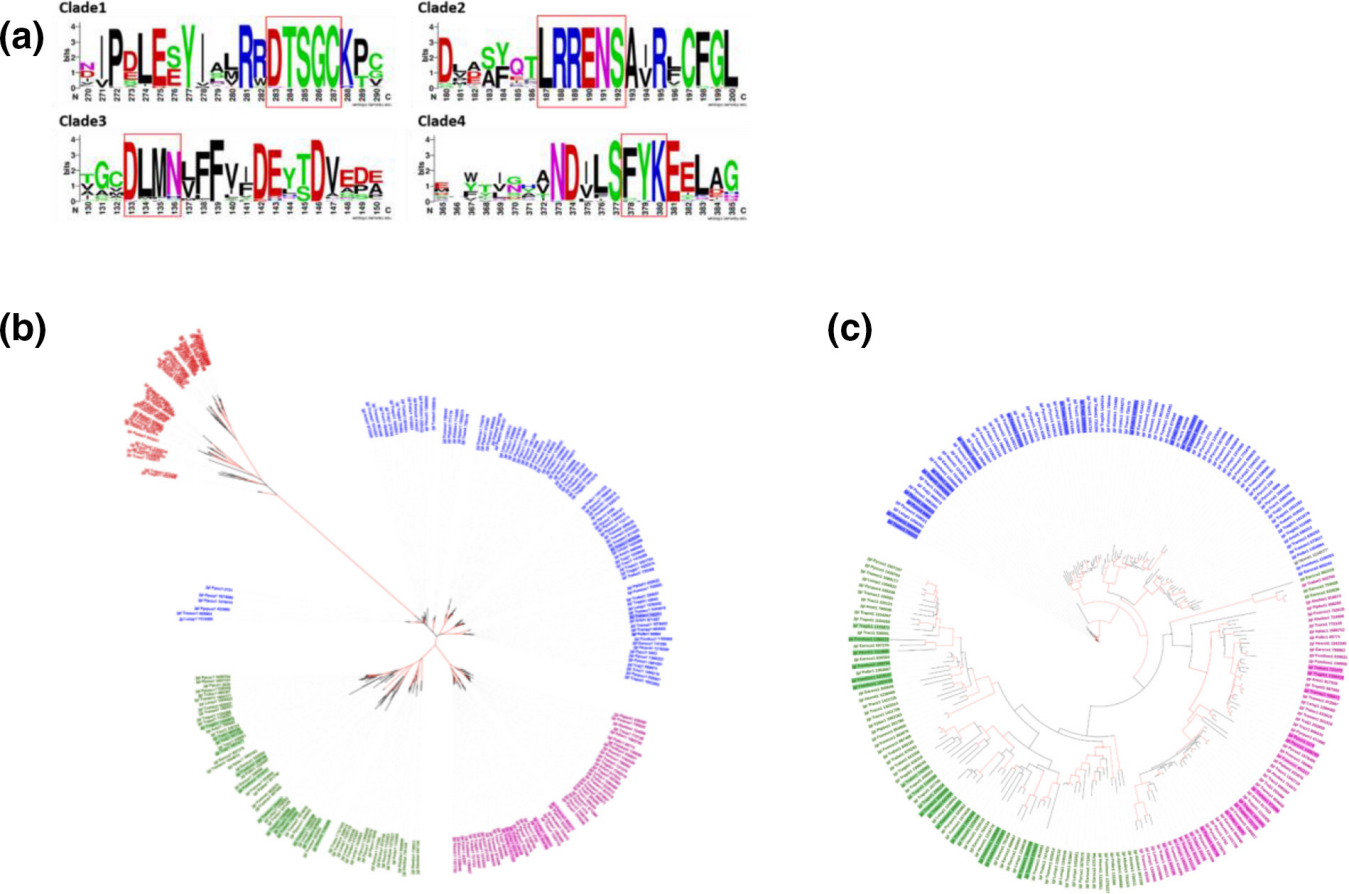
Refined assignment of a predicted type of cyclization to the sesquiterpene cyclases of 24 Polyporales species. **a**) WebLogo representation of the conserved motifs frequently found in STC sequences from each clade and totally absent in the sequences from the other clades. **b**) RAxML phylogenetic tree of Polyporales STC from clade 1 (blue), clade 2 (pink), clade 3 (green) and clade 4 (red). **c**) RAxML phylogenetic tree of Polyporales STC from clade 1 (blue), clade 2 (pink) and clade 3 (green). The previously unclassified STC that were reassigned to a clade according to the presence of the corresponding conserved motifs are highlighted with a coloured background. The red branches have bootstrap values>70.

To improve the categorization of the unclassified sequences, we searched for signature motifs specific of each of the four clades. For this purpose, we used the MERCI tool, which identifies the motifs that are enriched in a set of sequences and totally absent from the others [29]. Using MERCI on the sets of classified sequences, we identified the motif ‘DTSGC’ in 95 % of the clade 1 sequences, ‘LRRENS’ in 100 % of the clade 2 sequences, ‘DLMN’ in 77 % of the clade 3 sequences and finally ‘FYK’ in 92 % of the clade 4 sequences (Fig. 3a). Among the 88 sequences that were primarily ‘unclassified’, 24 (≈27 %) showed the conservation of one of those motifs and were reassigned accordingly to one of the four types, which resulted in a total of 89 Polyporales sequences classified in clade 1, 51 sequences classified in clade 2, 61 in clade 3, 44 in clade 4 and 64 still unclassified sequences. A phylogenetic analysis including the newly classified Polyporales STC sequences supported that the categorization of the sequences based on the conservation of the MERCI motifs was correct and that the sequences from clades 2, 3 and 4 were monophyletic (Fig. 3b, c). We observed that the Polyporales STC sequences classified in clade 1 are not monophyletic, indicating that great caution is necessary for the presupposition of the carbocation structure they produce. The unclassified sequences did not show monophyletic relationships, and were not further used to build STC HMM models (Fig. S1). The addition of ascomycete STC sequences in the phylogenetic analysis disrupted the monophyletic relationship between sequences from a same clade, suggesting that some of the ascomycete and basidiomycete sequences previously classified in the same clade had different evolutionary origins (Fig. S2).

### Confirmation of the STC activity for four newly identified enzymes

We tested the accuracy of the functional annotation and classification of four candidate STC genes from the Polyporales fungus *Leiotrametes menziesii*, one from each clade; Prot ID #671 455 (LmSTC1), #931 910 (LmSTC2), #920 014 (LmSTC3) and #1 051 559 (LmSTC4) (Fig. 2). After heterologous expression in *E. coli *, the four tested enzymes were active on (*E*,*E*)-farnesyl diphosphate (FPP) and produced one predominant reaction product (Fig. S3). LmSTC3 had promiscuous activity on GPP (Fig. S4). None of the four enzymes was active on GGPP (Fig. S5). Using GC-MS, we identified δ-cadinol as the reaction product of LmSTC1 (94 % match), as confirmed by NMR in another study (LmSTC1 was named STS-1*_Lm_* in [34, 34]; Fig. S6). We identified γ-cadinene as the reaction product of LmSTC2 (99 % match), and dauca-4(11),8-diene as the reaction product of LmSTC4 (99 % match). The reaction product for LmSTC3 was identified by RMN as Δ−6 protoilludene (Fig. S7, S8). The cyclic structure of the products was consistent with the expected primary cyclization of the FPP carbocation for each clade (Table 2; [17, 25, 35, 36]).

**Table 2.** Products of individual sesquiterpene cyclases from *Leiotrametes menziesii*

Clade	Clade 1	Clade 2	Clade 3	Clade 4
**enzyme**	LmSTC1	LmSTC2	LmSTC3	LmSTC4
**product**	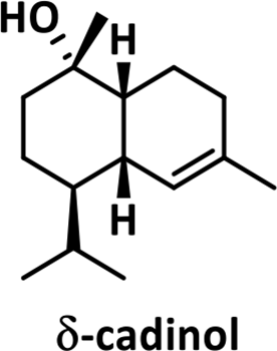	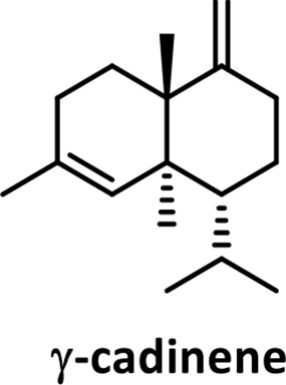	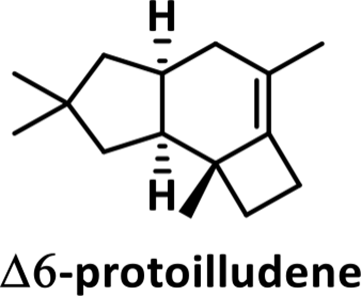	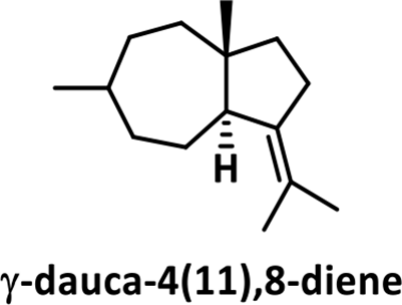
**primary cyclization**	1,10-cyclization of the (2*E*,6*E*)-FPP carbocation [36]	1,10-cyclization of the (3*R*)-NPP carbocation [35]	1,11 cyclization of the (2*E*,6*E*)-FPP carbocation [25]	1,7 cyclization of the (3*R*)-NPP carbocation [17]

### Generation of first generation-HMM models from Polyporales STC sequences

We used the sets of Polyporales sequences assigned to each clade to build first generation-HMM models. A search for these four HMM models on 656 fungal genomes available on Mycocosm retrieved a total of 5464 candidate STC genes. The genes retrieved by only one HMM model (threshold e^−25^) were assigned to the corresponding clade whereas the genes retrieved by the HMM models of different clades were assigned to the clade of the HMM model giving the highest score. This resulted in 1842 candidate STC classified in clade 1 (34 %), 1389 STC classified in clade 2 (25 %), 634 STC classified in clade 3 (12 %), and 1599 STC classified in clade 4 (29 %; Table 3). We tested the accuracy of the HMM models on a subset of characterized STC that were not used to build the first generation-HMM models (Table S4). All the previously characterized STC from clade 1, clade 2 and clade 4 were detected and assigned to the right clade. Among the eight characterized STC from clade 3, four were wrongly assigned to clade 1 or clade 2.

**Table 3.** Counts of sesquiterpene cyclase genes identified from 656 fungal genomes

	Counts of candidate STC genes	
Classification	First generation-HMM models	Second generation HMM-models
Clade 1	1842	1365
Clade 2	1389	878
Clade 3	634	1698
Clade 4	1599	1664
Ascomycota	1452	1407
Basidiomycota	4010	4194
Mucoromycota	0	1
Zoopagomycota	2	3
**Total**	**5464**	**5605**

### Generation of second generation-HMM models for fungal STC

To improve the assignment of candidate STC to the clades, we used the 100 sequences having the highest HMM scores for each model to build second generation-HMM models. A total of 5605 candidate STC genes were identified in the 656 fungal genomes using the second generation-HMM models, among which 24 % were classified in clade 1, 16 % in clade 2, 30 % in clade 3 and 30 % in clade 4 (Table 3). The characterized basidiomycete STC that were assigned to wrong clades with the first generation-HMM models were reassigned to the correct clade when using the second generation-HMM models (Table S4). We concluded that the second generation-HMM models were accurate for the prediction of the cyclization type of basidiomycete STC.

On the contrary, the models were less reliable for ascomycete sequences. For example, Ffsc4 was retrieved by the HMM models, but it was erroneously attributed to clade 1 (Table S4). Ffsc6 was not retrieved by any of the four HMM models, possibly due to the lack of phylogenetic relationship with STC sequences from the four clades analysed here (Fig. S2). We further tested the accuracy of the HMM models for the identification of Ascomycota STC on a set of 13 characterized STC for which the full-length coding sequence was properly annotated on Mycocosm. The four second generation-HMM models retrieved six Ascomycota STC out of the 13 (Table S5). These results show that the HMM models defined in this study can only identify a portion of the Ascomycota STC and can not be used to predict the cyclization type of Ascomycota STC.

Among the candidate STC genes found by second generation-HMM profiles, 22 % (1237 proteins) had a Pfam domain related to terpene synthase metal binding domain (PF03936) and 22 % (1437 proteins) had Pfam domains related to trichodiene synthase (PF06330). Yet, 52 % (4413 genes) did not have any Pfam annotation (Table 4). Interestingly, 45 % of the sequences had the new PF19086 domain only, indicating that this recent Pfam domain for C-terminal metal binding considerably enhanced the prediction for terpene synthases. The presence of a prenyltransferase Pfam domain (PF00348) in some of the newly identified STC can be explained by prenyltransferases featuring a DDxxD motif for binding divalent metal ions and sharing some sequence similarity with class I terpene synthases [1].

**Table 4.** Distribution of candidate STC and Pfam domains across the four clades

	Clade 1	Clade 2	Clade 3	Clade 4
**PF00348 -** Polyprenyl synthase	17	26	0	0
**PF03936 -** Terpene synthase family, metal binding domain	561	147	529	0
**PF06330 -** Trichodiene synthase (TRI5)	1	1	0	1435
**PF19086 only** - Terpene synthase family 2, C-terminal metal binding	759	675	1069	5
**Other Pfam**	0	0	2	5
**No Pfam**	27	29	98	219
Ascomycota (420 genomes analysed)	368	258	271	510
Basidiomycota (233 genomes analysed)	996	617	1427	1154
Mucoromycota (one genome analysed)	1	0	0	0
Zoopagomycota (two genomes analysed)	0	3	0	0

In total, 75 % of the candidate STC were retrieved from basidiomycete genomes, 25 % from ascomycetes and four genes from early diverging fungi (three genomes analysed; Table S6). An average of 17±10 STC genes per genome was found in basidiomycetes compared to 4±3 STC genes per genome in ascomycetes. Considering the fact that the initial HMM models were built from Polyporales sequences, we wondered if the HMM models could be biassed and better match basidiomycete sequences. We constructed four ascomycetes HMM models using the top 100 ascomycete sequences having the highest HMM scores from each clade. A search for candidate STC in 656 genomes using these Ascomycota-derived HMMs did not allow the identification of additional candidate STC.

### Tandem duplications have contributed to increased STC portfolios in Basidiomycota

We analysed the occurrence of STC gene duplications in Basidiomycota genomes. We considered genes were in tandem when a second copy was located among the four genes upstream or downstream a first copy on the same scaffold. We observed that 25.4 % of the STC genes in basidiomycete genomes were organized in tandem repeats. This result suggested that tandem duplications have played major roles in the expansion of the repertoires of STC genes in basidiomycetes. Yet, following duplication, each gene may evolve in several ways, retaining the same function as its ancestral copy, gaining a new function or becoming a nonfunctional gene [37]. Here we observed that 85 % of the STC genes organized in tandem repeat were grouped in the same clade, and consequently were predicted to initiate the same cyclization mechanism, and 15 % were grouped in different clades and predicted to initiate different cyclization mechanism. However, STC with slight protein sequence modifications might generate different final terpene products [38] and further studies are necessary to assess the diversity of terpenes produced by tandem genes. In addition, gene duplications might lead to the diversification of gene expression profiles, which can now be investigated.

## Conclusion

The HMM models we have defined here allowed the attribution of a predicted cyclization type for 4194 candidate STC from basidiomycete genomes. Previous analyses and our work suggest that, in the vast majority of cases, fungal STC catalyse the production of one major sesquiterpene product and have limited promiscuity. It was proposed that the fungal STC that grouped into a same phylogenetic clade catalysed the formation of primary carbocations with a same skeleton structure [15, 19, 25, 26]. Our results on four newly identified STC from the fungus *L. menziesii* supported this hypothesis. However, different cyclization paths may lead to the formation of a same terpene product and the identification of the intermediate products would be required for definitive demonstration [39]. Moreover, we observed that the introduction of newly identified STC sequences questioned the monophyletic structure of the previously described clade 1.

The HMM models proposed here were primarily based on sets of curated STC sequences identified from Basidiomycota genomes. The use of these HMM models on other fungi is only relevant for the identification of sequences that 1) are phylogenetically related to the Basidiomycota sequences from the same clade, or 2) share protein sequence features, possibly acquired through convergent evolution. As a consequence, some of the Ascomycota STC genes can be missed by these HMM models. Indeed, the HMM models retrieved six out of the 13 characterized STC we selected to test the accuracy on Ascomycota genomes. More studies will be necessary to define additional HMM models in order to cover the overall diversity of fungal sequences, in particular from Ascomycota and from phylogenetic clades additional to the four clades analysed here.

We observed that basidiomycete genomes contained more STC genes than that of ascomycetes. These findings were in line with previous studies that suggested Basidiomycota rely mostly on terpenoids as their predominant natural products, while Ascomycota have evolved a different arsenal of natural products, with the predominant production of polyketides (PK) and non-ribosomal peptides (NRP) [40]. We identified only a few genes in early diverging non-dikarya fungi, which is consistent with only triterpene and tetraterpene synthase found by other authors [41, 42].

Undoubtedly, more fungal enzymes will be characterized in the future, and their phylogenetic analysis will provide enlarged sets of phylogenetically related enzymes leading to a same cyclic primary FPP carbocation. Such enzyme sets will allow refining the HMM models for each clade. The improvement in our ability to predict the structure of the primary carbocations will enhance refined comparisons of fungal sesquiterpenomes and will considerably accelerate the discovery of new natural products through minimizing the re-discovery of already known molecules.

## Supplementary Data

Supplementary material 1
