# Supplementary material for: An HMM approach expands the landscape of sesquiterpene cyclases across the kingdom Fungi
Source: Microb Genom. 2023 Apr 19;9(4):mgen000990. doi: 10.1099/mgen.0.000990 (PMC10210940; doi:10.1099/mgen.0.000990)
Supplement: Supplementary material 1 [file mgen-9-990-s001.pdf]

Supplementary Information

An HMM approach expands the landscape of sesquiterpene cyclases across the kingdom Fungi.

Hayat Hage[1,2], Julie Couillaud[3,4] , Asaf Salamov[5], Margot Loussouarn-Yvon[1], Fabien Durbesson[6], Elena Ormeño[7], Sacha Grisel[1], Katia Duquesne[3], Renaud Vincentelli[6], Igor Grigoriev[5,8,9], Gilles Iacazio[3], Marie-Noëlle Rosso[1].

Author affiliations : [1]INRAE, Aix Marseille Univ, UMR1163, Biodiversité et Biotechnologie Fongiques, Marseille, France ; [2]Present Address: Bioaster, Lyon, France ; [3]Aix Marseille Univ, CNRS, Centrale Marseille, iSm2, Marseille, France ; [4]Present Address: Systems and Synthetic Biology Division, Department of Biology and Biological Engineering, Chalmers university of Technology, Gothenburg, Sweden; [5]US Department of Energy Joint Genome Institute, Lawrence Berkeley National Laboratory, Berkeley, CA 94720, USA; [6]AFMB, UMR CNRS 7257, USC 1408, Marseille, France ; [7]CNRS, Aix Marseille Univ, IRD, Avignon Univ, IMBE, Marseille, France; [8]Environmental Genomics and Systems Biology, Lawrence Berkeley National Laboratory, Berkeley, CA 94720, USA; [9]Department of Plant and Microbial Biology, University of California Berkeley, Berkeley, CA, USA

Content

Figure S1. RAxML phylogenetic tree of Polyporales STC with ambiguous classification (black), and from clade 1 (blue), clade 2 (pink), clade 3 (green), and clade 4 (red).

Figure S2. Phylogenetic tree of STC sequences. **Figure S3.** Reaction products of individual sesquiterpene cyclases from *Leiotrametes menziesii* on farnesyl diphosphate.

Figure S4. Reaction products of individual sesquiterpene cyclases from *Leiotrametes menziesii* on geranyl diphosphate.

Figure S5. Reaction products of individual sesquiterpene cyclases from *Leiotrametes menziesii* on geranylgeranyl diphosphate.

Figure S6. NMR data of δ-cadinol (^{1}H 600MHz, ^{13}C 150 MHz, solvent $CDCl_3$).

Figure S7. NMR data of Δ6-protoilludene (^{1}H 600MHz, ^{13}C 150 MHz, solvent $CDCl_3$).

Figure S8. Spectrum of the reaction product of LmSTC3 on FPP analyzed by 1H- **(a)** and 13C- **(b)** NMR.

Table S1. List of the fungal STC used in this study and for which the chemical structure of the reaction product on FPP was characterized.

Table S2. List of the 24 Polyporales genomes used in this study.

Table S3. Counts of blastp hits retrieved by each STC query sequence from each of the 24 Polyporales genomes.

Table S4. Assignment of characterized STC to each clade, based on 1st and 2nd generation-HMM models.

Table S5. Accuracy of the four 2nd generation- HMM models for retrieval of characterized STC sequences from Ascomycota genomes.*

Table S6. List of candidate STC identified in non-dikarya fungi and associated pfam and InterPro domains.

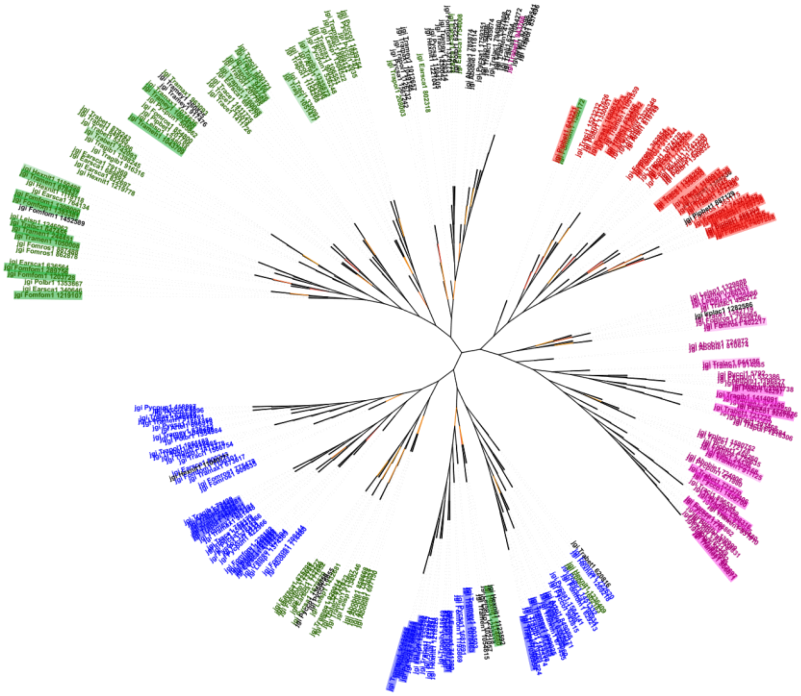

Figure S1. RAxML phylogenetic tree of Polyporales STC with ambiguous classification (black), and from clade 1 (blue), clade 2 (pink), clade 3 (green), and clade 4 (red). The branches with bootstrap values > 70 are indicated with purple disks.

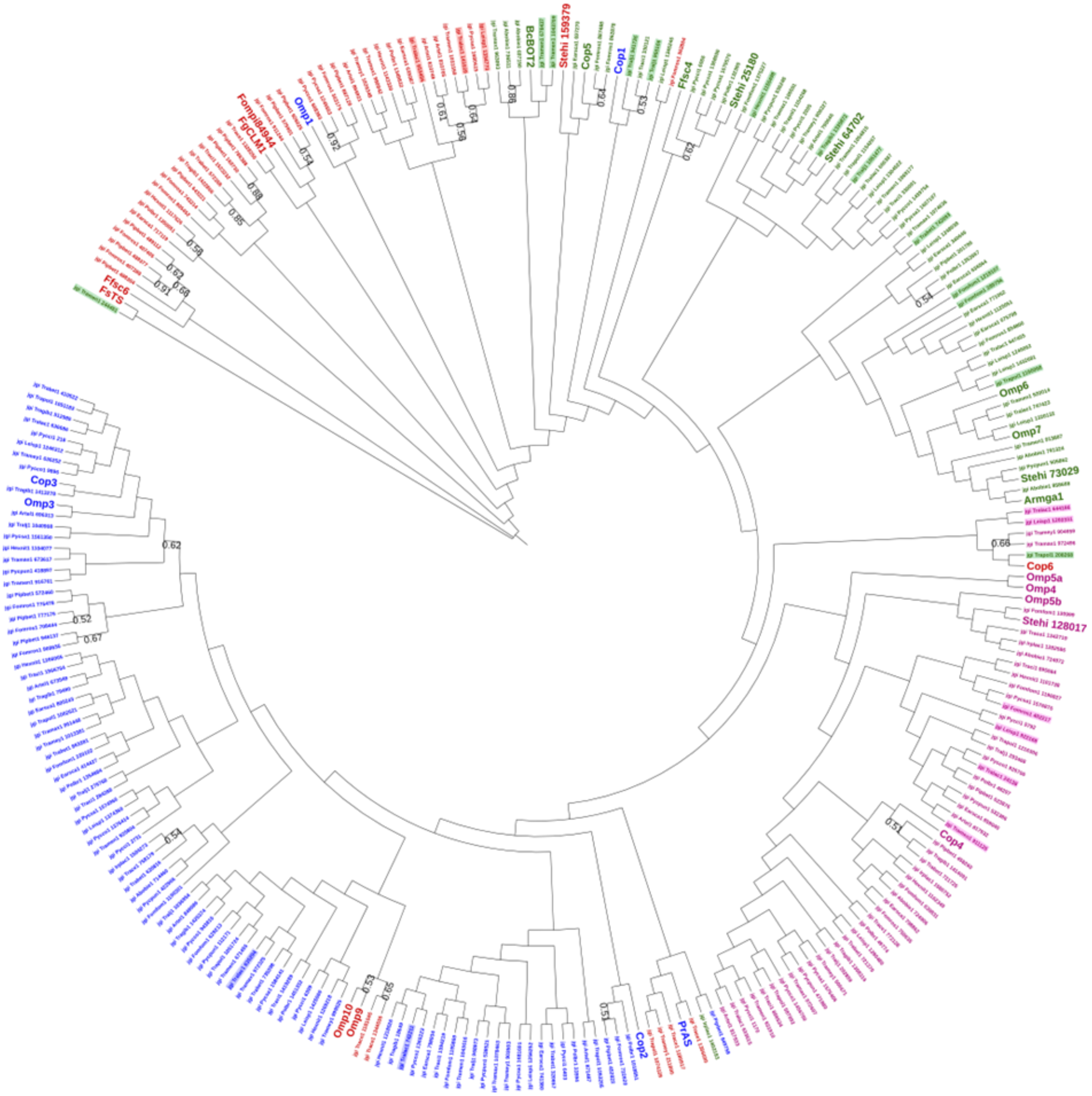

Figure S2. Phylogenetic tree of STC sequences. The tree includes the Polyporales sequences classified according to Fprotdist similarity (colored in blue for clade 1, pink for clade 2, grenn for clade 3 and red for clade 4), the sequences classified according to the MERCI motifs (with a colored background) and characterized STC (larger font size).

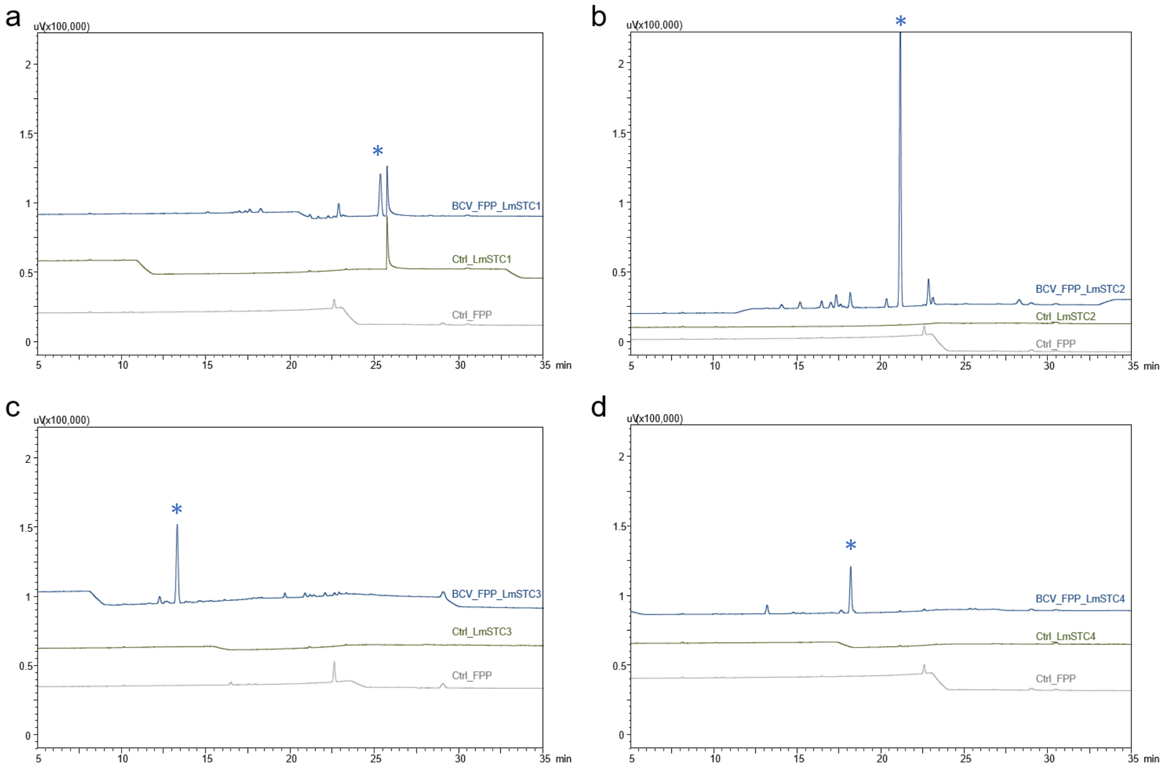

Figure S3. Reaction products of individual sesquiterpene cyclases from *Leiotrametes menziesii* on farnesyl diphosphate.

The reaction products on FPP were analyzed by gas chromatography for *Lm*STC1 **(a)**, *Lm*STC2 **(b)**, *Lm*STC3 **(c)** and *Lm*STC4 **(d)**. BCV: Bioconversion products; Ctrl_enzyme: the FPP was omitted; Ctrl_FPP: the enzyme was omitted. The main reaction product is indicated with an asterisks.

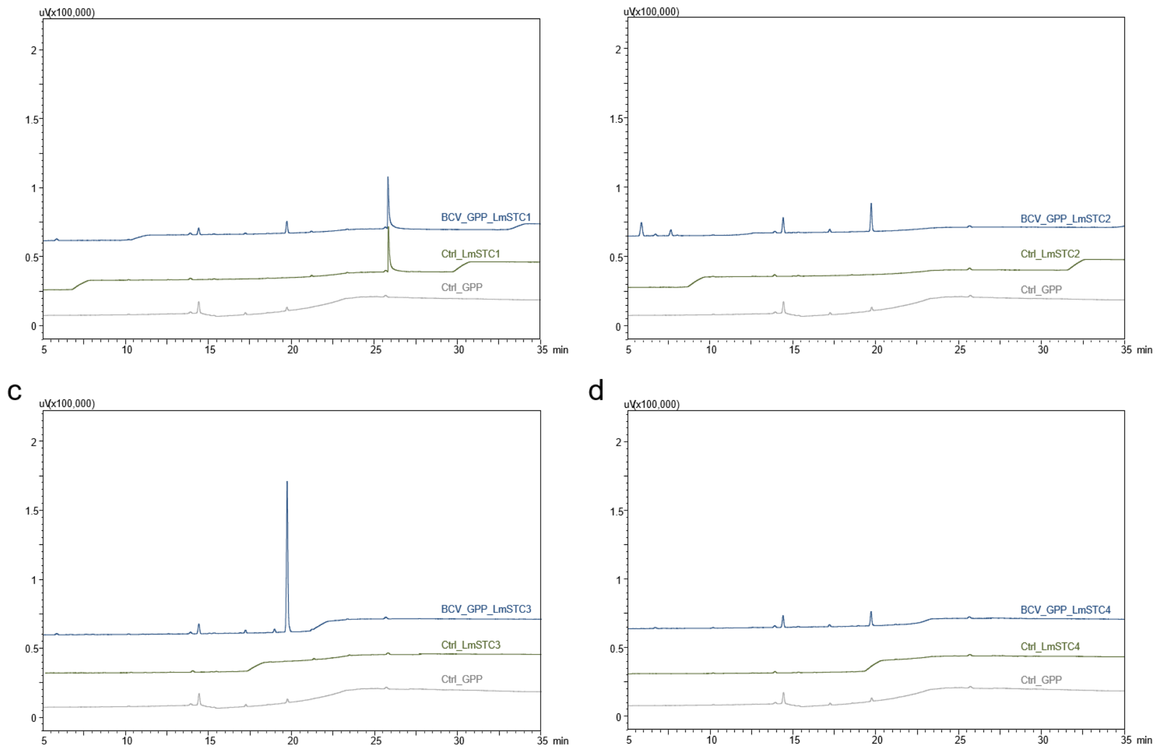

Figure S4. Reaction products of individual sesquiterpene cyclases from *Leiotrametes menziesii* on geranyl diphosphate.

The reaction products on GPP were analyzed by gas chromatography for *Lm*STC1 **(a)**, *Lm*STC2 **(b)**, *Lm*STC3 **(c)** and *Lm*STC4 **(d)**. BCV: Bioconversion products; Ctrl_enzyme: the GPP was omitted; Ctrl_GPP: the enzyme was omitted.

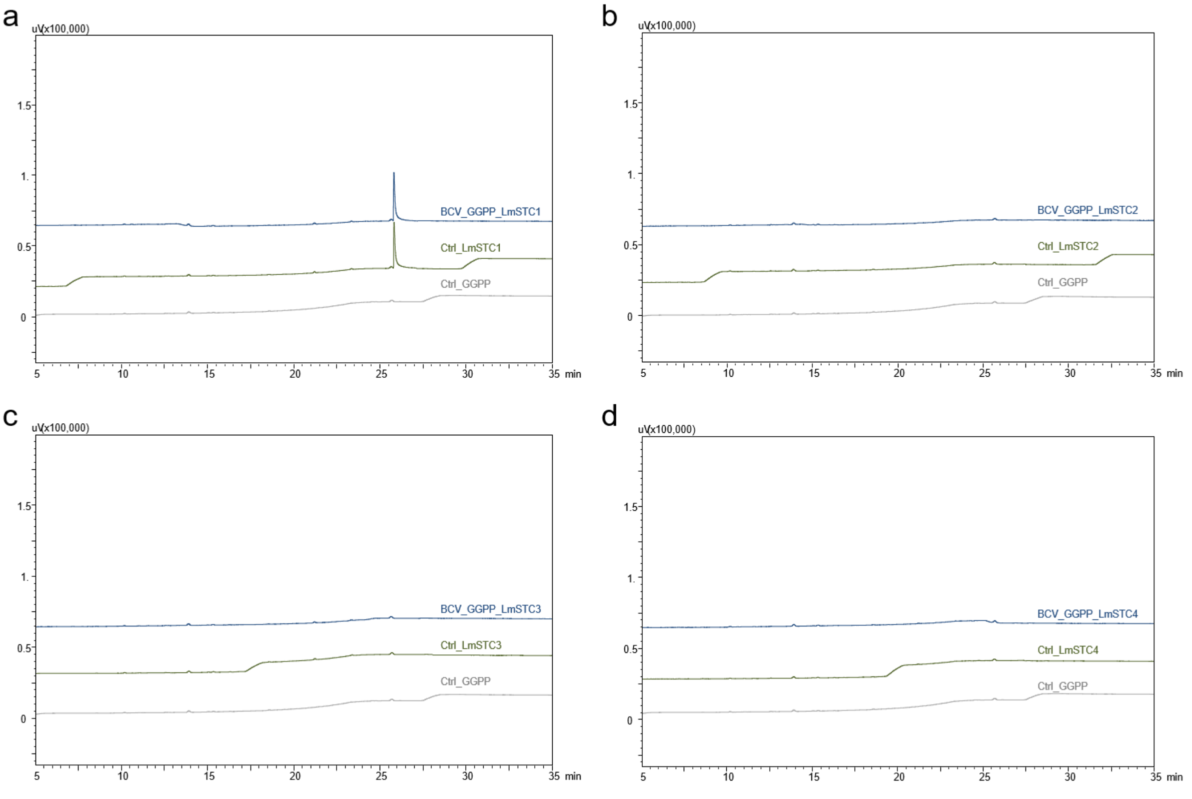

Figure S5. Reaction products of individual sesquiterpene cyclases from *Leiotrametes menziesii* on geranylgeranyl diphosphate.

The reaction products on GPP were analyzed by gas chromatography for *Lm*STC1 **(a)**, *Lm*STC2 **(b)**, *Lm*STC3 **(c)** and *Lm*STC4 **(d)**. BCV: Bioconversion products; Ctrl_enzyme: the GGPP was omitted; Ctrl_GGPP: the enzyme was omitted.

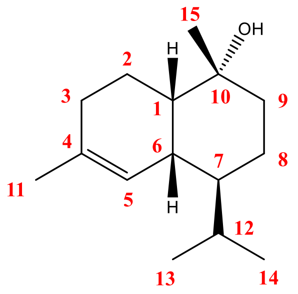

		This study	Ref S2	This study	Ref S2	This study	Ref S2
# C		δ (^{1}H) ppm	δ (^{1}H) ppm	δ (^{13}C) ppm	δ (^{13}C) ppm	*J* (Hz)	*J* (Hz)
1	CH	1.60 (1H, m)	1.60 (1H, m)	45.7	45.55		
2	CH_2	1.90 (1H, m)<br>1.56 (1H, m)	1.89 (1H, m)<br>1.58 (1H, m)	18.6	18.52		
3	CH_2	1.99 (2H, m)	1.99 (2H)	31.3	31.14		
4	C			134.2	134.37		
5	CH	5.62 (1H, dq)	5.51 (1H, m)	124.8	124.61	5.1, 1.7	
6	CH	2.02 (1H, m)	2.02 (1H, d)	36.9	36.79		
7	CH	1.31 (1H, m)	1.30 (1H)	44.2	44.10		
8	CH_2	1.50 (1H, m)<br>1.10 (1H, qd)		21.7	21.54	13.1, 4.3	
9	CH_2	1.57 (1H, m)<br>1.51 (1H, m)	1.56 (1H, t)<br>1.52 (1H, t)	35.3	35.34		
10	C			72.7	72.58		
11	CH_3	1.66 (3H, brs)	1.66 (3H)	23.8	23.64		
12	CH	1.97 (1H, m)	1.97 (1H, m)	26.8	26.42		
13	CH_3	0.88 (3H, d)	0.89 (3H, d)	15.6*	21.69	6.9	
14	CH_3	0.81 (3H, d)	0.81 (3H, d)	21.8*	15.33	6.9	
15	CH_3	1.30 (3H, s)	1.29 (3H)	28.2	27.97		

Figure S6. NMR data of δ-cadinol (^{1}H 600MHz, ^{13}C 150 MHz, solvent $CDCl_3$).

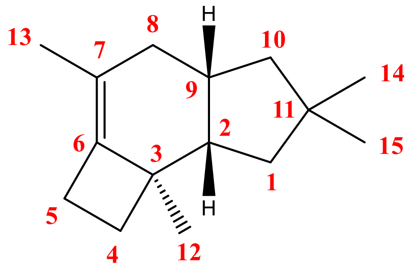

		This study	Ref S1	This study	Ref S1	This study	Ref S1
# C		δ (^{1}H) ppm	δ (^{1}H) ppm	δ (^{13}C) ppm	δ (^{13}C) ppm	*J* (Hz)	*J* (Hz)
1	CH_2	1.28 (1 H, dd)<br>1.35 (1 H, ddd)	1.29 (1 H, dd) α<br>1.35 (1 H, ddd) β	41.2	41.1	12.0, 11.2<br>12.5, 8.0, 1.8	11, 12<br>2, 8, 12
2	CH	2.17 (1 H, ddd)	2.16 (1 H, ddd) β	47.2	47.1	11.0, 11.0, 8.0	8, 11
3	C			45.9	45.7		
4	CH_2	1.74 (1H, br. q)<br>1.81 (1H, ddd)	1.74 (1 H, m) β<br>1.81 (1 H, ddd) α	37.0	36.9	9.2<br>9.9, 9.5, 3.3	3, 9, 11
5	CH_2	2.53 (1 H, m)<br>2.73 (1 H, m)	2.53 (1 H, m) α<br>2.72 (1 H, m) β	25.6	25.5		
6	C			141.9	141.8		
7	C			123.2	123.1		
8	CH_2	1.65 (1 H, m)<br>1.85 (1 H, dd)	1.65 (1 H, m) α<br>1.85 (1 H, dd) β	34.3	34.2	14.4, 6.8	7, 14
9	CH	2.34 (1 H, qt)	2.34 (1 H, qt) β	40.7	40.6	10.7, 6.9	7, 11
10	CH_2	0.97 (1 H, m)<br>1.53 (1 H, ddd)	0.96 (1 H, m) α<br>1.53 (1 H, ddd) β	48.8	48.6	12.3, 7.4, 1.9	2, 7, 10
11	C			39.4	39.2		
12	CH_3	1.06 (3H, s)	1.06 (3H, s)	20.6	20.5		
13	CH_3	1.57 (3H, s)	1.57 (3H, br. s)	17.5	17.4		
14	CH_3	0.93 (3H, s)	0.93 (3H, s)	27.6	27.5		
15	CH_3	1.04 (3H, s)	1.04 (3H, s)	30	29.8		

Figure S7. NMR data of Δ6-protoilludene (^{1}H 600MHz, ^{13}C 150 MHz, solvent $CDCl_3$)

a

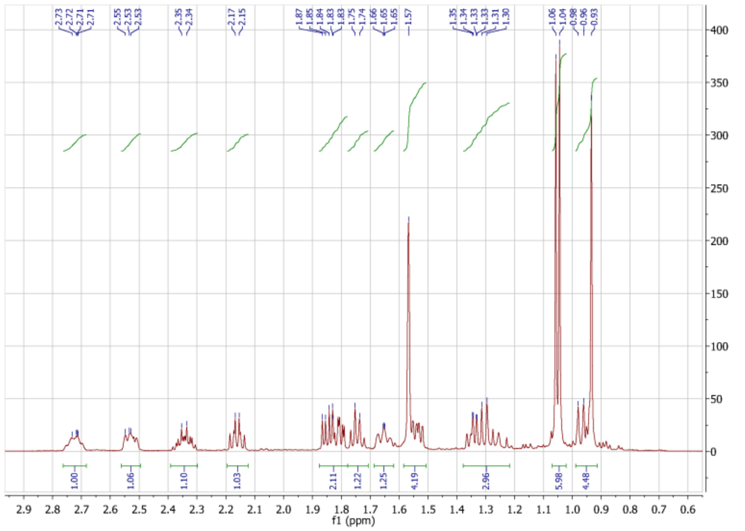

b

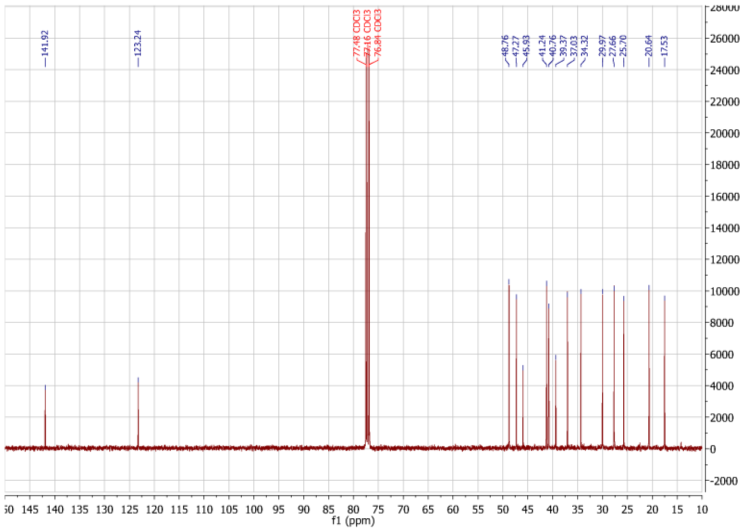

Figure S8. Spectrum of the reaction product of LmSTC3 on FPP analyzed by 1H- **(a)** and 13C- **(b)** NMR.

Table S1. List of the fungal STC used in this study and for which the chemical structure of the reaction product on FPP was characterized.
[1] Agger et al., Mol Microbiol. 2009; [2] Wawrzyn et al., Chem Biol., 2012 ; [3] Caruthers et al., J Biol Chem, 2000; [4] Felicetti et al., J Am Chem Soc., 2004; [5] Quin et al., Chembiochem, 2013; [6] Engels et al., J Biol Chem, 2011; [7] Pinedo et al., ACS Chem Biol., 2008; [8] Wang et al., J Am Chem Soc, 2009; [9] Brock et al., Chembiochem, 2011); [10] Brock et al., Chembiochem, 2013; [11] Rynkiewicz et al., Proc Natl Acad Sci USA, 2001; [12] Cane et al., Arch Biochem Biophys., 1993; [13] McCormick et al., Appl Environ Microbiol, 2010. Pfam and InterPro domains were identified using InterProscan; Paysan-Lafosse et al., Nucleic Acids Res., 2022.

Type of cyclization	Phylum	order	species	Gene name	Mycocosm Protein Id	GenBankID	main reaction products	used as query in blast searches	references	Pfam domains	InterPro domains
Clade 1 1,10-cyclization of the (2*E*,6*E*)-FPP carbocation	Basidiomycota	Agaricales	*Coprinopsis cinereus*	Cop1	jgi|Copci1|3117	XP001832573	germacrene A	No	[1]	PF19086	IPR008949
	Basidiomycota	Agaricales	*Coprinopsis cinereus*	Cop2	jgi|Copci_AmutBmut1|443805	XP001836556	germacrene A	yes	[1]	PF19086	IPR008949; IPR034686
	Basidiomycota	Agaricales	*Coprinopsis cinereus*	Cop3	jgi|Copci_AmutBmut1|124670	XP001832925	α-muurolene, germacrene A	No	[1]	PF19086	IPR008949; IPR034686
	Basidiomycota	Agaricales	*Omphalotus olearius*	Omp1	jgi|Ompol1|1311		α-muurolene	No	[2]	PF19086	IPR008949; IPR034686
	Basidiomycota	Agaricales	*Omphalotus olearius*	Omp3	jgi|Ompol1|4636		α-muurolene, germacrene A	yes	[2]	PF19086	IPR008949; IPR034686
	Ascomycota	Eurotiomycete	*Penicilum roqueforti*	PrAS	jgi|Penro1|5468	Q03471	aristolochene	No	[3],[4]	PF19086	IPR008949; IPR034686
Clade 2 1,10-cyclization of the (3*R*)-NPP carbocation	Basidiomycota	Russulales	*Stereum hirsutum*		jgi|Stehi1|128017		d-Cadinene	yes	[5]	PF19086	IPR008949; IPR034686
	Basidiomycota	Agaricales	*Coprinopsis cinereus*	Cop4	jgi|Copci_AmutBmut1|30510	XP001836356	δ-Cadinene, cubedol, germacrene D	No	[1]	PF19086	IPR008949; IPR034686
	Basidiomycota	Agaricales	*Omphalotus olearius*	Omp4	jgi|Ompol1|1447		δ-Cadinene	No	[2]	PF19086	IPR008949; IPR034686
	Basidiomycota	Agaricales	*Omphalotus olearius*	Omp5a	jgi|Ompol1|2392		epi-zonarene, γ-cadinene	yes	[2]	PF19086	IPR008949; IPR034686
	Basidiomycota	Agaricales	*Omphalotus olearius*	Omp5b	jgi|Ompol1|2393		γ-cadinene	No	[2]	PF19086	IPR008949; IPR034686
Clade 3 1,11 cyclization of the (2*E*,6*E*)-FPP carbocation (trans-humulyl-carbocation)	Basidiomycota	Russulales	*Stereum hirsutum*		jgi|Stehi1|73029		Δ6-protoilludene	No	[5]	PF19086	IPR008949; IPR034686
	Basidiomycota	Russulales	*Stereum hirsutum*		jgi|Stehi1|64702		Δ6-protoilludene	No	[5]	PF19086	IPR008949; IPR034686
	Basidiomycota	Russulales	*Stereum hirsutum*		jgi|Stehi1|25180		Δ6-protoilludene	No	[5]	PF19086	IPR008949
	Basidiomycota	Agaricales	*Omphalotus olearius*	Omp6	jgi|Ompol1|4774		Δ6-protoilludene	yes	[2]	PF19086	IPR008949; IPR034686
	Basidiomycota	Agaricales	*Omphalotus olearius*	Omp7	jgi|Ompol1|2271		Δ6-protoilludene	No	[2]	PF19086	IPR008949; IPR034686
	Basidiomycota	Agaricales	*Coprinopsis cinereus*	Cop5	-	XP001834007	pentalenene	No	[1]	PF19086	IPR008949
	Basidiomycota	Agaricales	*Armilaria gallica*	Armga1	jgi|Armga1|1000221	AGR34199	Δ6-protoilludene	No	[6]	PF19086	IPR008949; IPR034686
	Ascomycota	Leotiomycete	*Botrytis cinerea*	BcBOT2	-	AAQ16575	presilphiperfolan-8b-ol	yes	[7]; [8]	PF19086	IPR008949; IPR034686
	Ascomycota	Sordariomycete	*Fusarium fujikuroi*	Ffsc4	jgi|Fusfu1|11322	CCP20071	koraiol	No	[9]; [10]	PF19086	IPR008949; IPR034686
Clade 4 1,6 or 1,7 cyclization of the (3*R*)-NPP carbocation	Basidiomycota	Russulales	*Stereum hirsutum*		jgi|Stehi1|159379		β-barbatene	No	[5]		IPR008949; IPR024652
	Basidiomycota	Agaricales	Coprinopsis cinereus	Cop6	jgi|Copci1|3094		α-cupranene	No	[1]		IPR008949; IPR024652
	Basidiomycota	Agaricales	*Omphalotus olearius*	Omp9	jgi|Ompol1|3258		a/b-barbatene	No	[2]		IPR008949; IPR024652
	Basidiomycota	Agaricales	*Omphalotus olearius*	Omp10	jgi|Ompol1|3981		daucene/trans-dauca-4(11),8-diene	yes	[2]		IPR008949; IPR024652
	Basidiomycota	Polyporales	*Fomitopsis pinicola*	Fompi84944	jgi|Fompi3|84944		α-cuprenene	yes	[2]		IPR008949; IPR024652
	Ascomycota	Sordariomycete	*Fusarium fujikuroi*	Ffsc6	jgi|Fusfu1|12876	CCP20072	(−)-α-acorenol	yes	[9]; [10]	PF19086	IPR008949
	Ascomycota	Sordariomycete	*Fusarium spordrichoides*	FsTS	-	AAN05035	trichodiene	No	[11], [12]	PF06330	IPR010458; IPR024652
	Ascomycota	Sordariomycete	*Fusarium graminearum*	FgCLM1	-	ACY69978	longiborneol	No	[13]	PF06330	IPR008949; IPR024652

Table S2. List of the 24 Polyporales genomes used in this study.

Species name	Strain CIRM-BRFM	Genome name	Genome NCBI ID
Abortiporus biennis	CIRM-BRFM 1778	Abobie1	JABYQP000000000
Artolenzites elegans	CIRM-BRFM 1663	Artel1	JABYQO000000000
Earliella scabrosa	CIRM-BRFM 1817	Earsca1	JABYQQ000000000
Fomes fomentarius	CIRM-BRFM 1821	Fomfom1	JABYQN000000000
Fomitopsis betulina	CIRM-BRFM 1772	Pipbet1	JABYQK000000000
Hexagonia nitida	CIRM-BRFM 1802	Hexnit1	JABYQM000000000
Irpex lacteus	CCBAS Fr. 238 617/93	Irplac1	JABYQL000000000
Leiotrametes lactinea	CIRM-BRFM 1664	Tralac1	JACDTU000000000
Leiotrametes menziesii	CIRM-BRFM 1781	Tramen1	JABYQH000000000
Leiotrametes sp.	CIRM-BRFM 1775	Leisp1	JABYMW000000000
Rhodofomes roseus	CIRM-BRFM 1785	Fomros1	JADCUA000000000
Trametes betulina	CIRM-BRFM 1801	Trabet1	JABYQU000000000
Trametes cingulata	CIRM-BRFM 1805	Traci1	JABYMY000000000
Trametes gibbosa	CIRM-BRFM 1770	Tragib1	JABYQJ000000000
Trametes ljubarskyi	CIRM-BRFM 1659	Tralj1	JABYMZ000000000
Trametes maxima	CIRM-BRFM 1813	Tramax1	JABYQI000000000
Trametes meyenii	CIRM-BRFM 1810	Tramey1	JABYQG000000000
Trametes polyzona	CIRM-BRFM 1798	Trapol1	JABYQF000000000
Trametopsis cervina	CIRM-BRFM 1824	Trace1	JABYMX000000000
Pycnoporus coccineus	CIRM-BRFM 310	Pycco1	NCSW00000000.1
Pycnoporus puniceus	CIRM-BRFM 1868	Pycpun1	VICQ00000000
Polyporus brumalis	CIRM-BRFM 1820	Polbr1	PTTP00000000
Pycnoporus cinnabarinus	CIRM-BRFM 137	Pycci1	GCA_000765035.1
Pycnoporus sanguineus	CIRM-BRFM 1264	Pycsa1	VOCM00000000

Table S3. Counts of blastp hits retrieved by each STC query sequence from each of the 24 Polyporales genomes.

Polyporales genomes used as database	STC sequences used as queries	Counts of blastp hits
Abobie1	BcBOT2	1
	Ffsc6	2
	Omp5a	8
	Omp6	11
	Stehi	10
	cop2	9
Artel1	BcBOT2	2
	Omp10	3
	Omp5a	11
	Omp6	8
	Stehi	6
	cop2	10
Earsca1	Fompi	2
	Omp10	3
	Omp5a	11
	Omp6	17
	Stehi	9
	cop2	10
Fomfom1	Fompi	1
	Omp5a	11
	Omp6	17
	Stehi	10
	cop2	13
Fomros1	BcBOT2	1
	Fompi	7
	Omp10	8
	Omp5a	9
	Omp6	10
	Stehi	7
	cop2	8

Polyporales genomes used as database	STC sequences used as queries	Counts of blastp hits
Pipbet1	Ffsc6	1
	Fompi	10
	Omp10	7
	Omp5a	9
	Omp6	8
	Stehi	10
	cop2	8
Polbr1	Ffsc6	1
	Omp10	2
	Omp5a	9
	Omp6	10
	Stehi	8
	cop2	8
Pycci1	Omp5a	8
	Omp6	10
	Stehi	7
	cop2	8
Pycco1	Ffsc6	2
	Fompi	2
	Omp10	1
	Omp5a	8
	Omp6	9
	Stehi	6
	cop2	9
Pycpun1	Ffsc6	1
	Fompi	1
	Omp10	2
	Omp5a	8
	Omp6	10
	Stehi	7
	cop2	11

Polyporales genomes used as database	STC sequences used as queries	Counts of blastp hits
Traci1	BcBOT2	2
	Ffsc6	1
	Fompi	1
	Omp10	2
	Omp5a	10
	Omp6	10
	Stehi	9
	cop2	9
Tragib1	Fompi	2
	Omp10	1
	Omp5a	10
	Omp6	11
	Stehi	11
	cop2	11
Tralac1	BcBOT2	2
	Omp10	2
	Omp5a	13
	Omp6	13
	Stehi	11
	cop2	10
Tralj1	Fompi	1
	Omp5a	11
	Omp6	11
	Stehi	11
	cop2	9
Tramax1	Ffsc6	1
	Fompi	1
	Omp10	1
	Omp5a	8
	Omp6	10
	Stehi	9
	cop2	10

Polyporales genomes used as database	STC sequences used as queries	Counts of blastp hits
Hexnit1	Omp10	2
	Omp5a	9
	Omp6	16
	Stehi	9
	cop2	11
Irplac1	BcBOT2	1
	Ffsc6	1
	Fompi	1
	Omp10	1
	Omp5a	6
	Omp6	5
	Stehi	5
	cop2	5
Leisp1	Ffsc6	1
	Fompi	2
	Omp10	1
	Omp5a	15
	Omp6	20
	Stehi	14
	cop2	15
Pycsa1	Fompi	1
	Omp10	1
	Omp5a	11
	Omp6	10
	Stehi	9
	cop2	12
Trabet1	Fompi	1
	Omp5a	12
	Omp6	14
	Stehi	12
	cop2	9

Polyporales genomes used as database	STC sequences used as queries	Counts of blastp hits
Tramen1	Ffsc6	1
	Fompi	2
	Omp10	1
	Omp5a	14
	Omp6	18
	Stehi	12
	cop2	15
Tramey1	Fompi	1
	Omp10	1
	Omp5a	9
	Omp6	11
	Stehi	9
	cop2	11
Trapol1	Fompi	3
	Omp5a	10
	Omp6	13
	Stehi	9
	cop2	13
Trace1	Fompi	6
	Omp10	2
	Omp5a	6
	Omp6	6
	Stehi	5
	cop2	7

Table S4. Assignment of characterized STC to each clade, based on 1st and 2nd generation-HMM models. Mis-assignments as compared to C. Schmidt-Dannert et al. (2014) are highlighted in bold.

STC	JGI ID	expected classification	Classification based on 1st generation HMM profiles	Classification based on 2nd generation HMM profiles
Cop2	jgi|Copci_AmutBmut1|443805	Clade 1	clade 1	clade 1
Cop1	jgi|Copci1|3117		clade 1	clade 1
Omp1	jgi|Ompol1|1311		clade 1	clade 1
Cop3	jgi|Copci_AmutBmut1|124670		clade 1	clade 1
Omp3	jgi|Ompol1|4636		clade 1	clade 1
Omp5a	jgi|Ompol1|2392	Clade 2	clade 2	clade 2
Omp5b	jgi|Ompol1|2393		clade 2	clade 2
Omp4	jgi|Ompol1|1447		clade 2	clade 2
Cop4	jgi|Copci_AmutBmut1|30510		clade 2	clade 2
Stehi 128017	jgi|Stehi1|128017		clade 2	clade 2
Ffsc4	jgi|Fusfu1|11322	Clade 3	**clade 1**	**clade 1**
Stehi 73029	jgi|Stehi1|73029		**clade 2**	clade 3
Armga1	jgi|Armga1|1000221		**clade 1**	clade 3
Stehi 64702	jgi|Stehi1|64702		clade 3	clade 3
Omp7	jgi|Ompol1|2271		clade 3	clade 3
Stehi 25180	jgi|Stehi1|25180		clade 3	clade 3
Omp6	jgi|Ompol1|4774		**clade1**	clade 3
Fompi 84944	jgi|Fompi3|84944	Clade 4	clade 4	clade 4
Stehi 159379	jgi|Stehi1|159379		clade 4	clade 4
Cop6	jgi|Copci1|3094		clade 4	clade 4
Omp9	jgi|Ompol1|3258		clade 4	clade 4
Omp10	jgi|Ompol1|3981		clade 4	clade 4
Ffsc6	jgi|Fusfu1|12876		not found	not found

Table S5. Accuracy of the four 2nd generation- HMM models for retrieval of characterized STC sequences from Ascomycota genomes.
[1] Brock, et al., Chembiochem : a European journal of chemical biology, 2013; [2] McCormick et al., Appl Environ Microbiol., 2010; [3] Proctor et al., Mol. Plant Microbe Interact., 1995; [4] Stajich et al., Nucleic Acids Res., 2011; [5] Wu et al., PLoS One, 2016; [6] Cheeseman et al., Nat. Commun., 2014; [7] Tijerino et al., Fungal Genet. Biol., 2011.

**Fungal species**	**gene**	**JGI ProtID**	**Retrieved with 2nd generation HMM models**	**Reference**
Fusarium fujikori	Ffsc4	Fusfu1|11322	no	[1]
Fusarium fujikori	Ffsc6	Fusfu1|12876	no	[1]
Fusarium graminearum	FgCLM1	Fusgr1|12023 (exon missing)	no	[2]
Fusarium graminearum	tri5	Fusgr1|4586	no	[3]
Fusarium graminearum	FGRAMPH1_01G21787	Fusgr1|13217	clade1	[4]
Fusarium graminearum	FGRAMPH1_01G25349	Fusgr1|8874	clade1	[4]
Fusarium graminearum	FGRAMPH1_01G04331	Fusgr1|2052	clade1	[4]
Fusarium graminearum	FGRAMPH1_01G13013	Fusgr1|4632	no	[4]
Hypoxylon sp.	CI4A-6706	HypCI4A_1|6706	clade2	[5]
Hypoxylon sp.	CI4A-322581	HypCI4A_1|322581	clade1	[5]
Hypoxylon sp. C	CO27-397991	HypCI4A_1|6706	clade2	[5]
Penicillium roqueforti	prAS	Penro1|5468	no	[6]
Trichoderma brevicompactum	tri5	Tribe|118137	no	[7]

Table S6. List of candidate STC identified in non-dikarya fungi and associated pfam and InterPro domains.

phylum	analyzed genomes	jgiID	protID	Pfam	InterPro
Zoopagomycota	*Basidiobolus meristosporus*	Basme2finSC	jgi|Basme2finSC|301341	PF19086	IPR008949; IPR034686
			jgi|Basme2finSC|304520	PF19086	IPR008949; IPR034686
Zoopagomycota	*Zoophthora radicans*	Zoorad1	jgi|Zoorad1|484271	PF19086	IPR008949; IPR034686
Mucoromycota	*Gigaspora rosea*	Gigro1	jgi|Gigro1|2026435	PF19086	IPR008949
			jgi|Gigro1|2151269	PF19086	IPR008949
			jgi|Gigro1|1892979	PF19086	IPR008949; IPR034686
			jgi|Gigro1|1965004	PF19086	IPR008949; IPR034686
